# Predictive value of tissue eosinophilia for dupilumab response in chronic rhinosinusitis with nasal polyps: A retrospective monocentric study

**DOI:** 10.1016/j.waojou.2025.101129

**Published:** 2025-10-09

**Authors:** Gian Marco Pace, Giulia Mari, Francesco Giombi, Michele Cerasuolo, Camilla Zimello, Luca Cerri, Giorgio Walter Canonica, Enrico Heffler, Giovanni Paoletti, Francesca Puggioni, Barbara Fiamengo, Silvia Uccella, Giuseppe Mercante, Giuseppe Spriano, Luca Malvezzi

**Affiliations:** a Department of Biomedical Sciences, Humanitas University, Via Rita Levi Montalcini 4, Pieve Emanuele, Milan, 20090 Italy; b Otorhinolaryngology Unit, IRCCS Humanitas Research Hospital, Via Manzoni 56, Rozzano, Milan, 20089 Italy; c Otorhinolaryngology Head & Neck Surgery Unit, Casa di Cura Humanitas San Pio X, Via Francesco Nava 31, Milan, 20159 Italy; d Personalized Medicine, Asthma and Allergy, IRCCS Humanitas Research Hospital, Via Manzoni 56, Rozzano, Milan, 20089 Italy; e Unit of Pathological Anatomy, IRCCS Humanitas Research Hospital, Via Manzoni 56, Rozzano, Milan, 20089 Italy

**Keywords:** Chronic rhinosinusitis, Nasal polyps, Dupilumab, Tissue eosinophilia, Patient-reported outcome

## Abstract

**Objective:**

To evaluate the predictive value of baseline tissue eosinophilic infiltration (cells/high-power field [HPF]) in determining clinical response to dupilumab in patients with chronic rhinosinusitis with nasal polyps (CRSwNP).

**Methods:**

This retrospective, single-center observational study included patients with severe, refractory CRSwNP treated with dupilumab between December 2020 and December 2024. Based on histopathological analysis of nasal polyp biopsies, patients were stratified into 2 groups according to tissue eosinophil density: <50 cells/HPF and ≥50 cells/HPF. Clinical response was assessed through patient-reported outcomes (SNOT-22) and objective measures including Nasal Polyp Score (NPS), Lund-Kennedy Score (LKS), and Lund-Mackay Score (LMS), evaluated at baseline and after 1, 3, and 12 months of treatment. Repeated-measures ANOVA was used to evaluate within-group and between-group differences over time.

**Results:**

Eighty-six patients were included in the analysis: 57 with low eosinophilic infiltration (<50 cells/HPF) and 29 with high infiltration (≥50 cells/HPF). Patients with high tissue eosinophilia showed significantly greater improvement in SNOT-22 scores across all time points (p = 0.045). No significant between-group differences were found in endoscopic (NPS, LKS) or radiologic (LMS) outcomes throughout the follow-up period.

**Conclusions:**

Dupilumab confirmed high clinical efficacy, rapid symptom improvement, and good tolerability in patients with CRSwNP. Higher tissue eosinophil counts were associated with greater symptom improvement, as measured by SNOT-22. These findings suggest a potential role for tissue eosinophilia as a predictive marker of clinical response.

## Introduction

Chronic rhinosinusitis with nasal polyps (CRSwNP) is a clinical phenotype of chronic rhinosinusitis, defined by persistent inflammation of the nasal and paranasal sinus mucosa lasting for ≥12 weeks, with bilateral nasal polyposis confirmed by endoscopic examination.^1^ The condition is frequently associated with a type 2 inflammatory endotype, characterized by elevated expression of interleukin (IL)-4, IL-5, and IL-13 in the inflammatory microenvironment, along with dense eosinophilic infiltration of the sinonasal mucosa.^2^ This immunologic profile has enabled the development of monoclonal antibodies targeting key mediators of type 2 inflammation.^3^, ^4^, ^5^, ^6^ Dupilumab is a fully human monoclonal antibody that inhibits IL-4 and IL-13 signaling through blockade of the IL-4 receptor alpha subunit (IL-4Rα), and has been approved for the treatment of CRSwNP inadequately controlled with intranasal corticosteroids and/or prior surgical interventions.^3^ Clinical trials have demonstrated that dupilumab significantly improves nasal obstruction, polyp size, olfactory function, and quality of life in patients with severe disease.^7^^,^^8^ Even though dupilumab demonstrated significant clinical benefits in CRSwNP patients, there remains a considerable degree of interindividual variability in treatment response. Remarkably, in the pooled analysis of the SINUS-24 and SINUS-52^3^ trials, approximately 10% of patients receiving dupilumab still required rescue systemic corticosteroids or functional endoscopic sinus surgery during the study period, highlighting the existence of a non-responder subset despite targeted biologic therapy.

While a number of factors have been explored to explain these differences in response, no clear or consistent predictors have been identified.^9^^,^^10^ Given the role of eosinophils as key effector cells in type 2 inflammation, peripheral blood eosinophilia has been proposed as a biomarker of this inflammatory endotype and has been associated with treatment outcomes in eosinophilic asthma.^11^ In CRSwNP, however, the evidence remains inconsistent.^12^^,^^13^ Post hoc analyses of the SINUS-52 trial indicate that baseline peripheral eosinophil counts may not reliably predict response to dupilumab.^3^ In contrast, tissue eosinophilia, quantified as the number of eosinophilic cells per high-power field (cells/HPF) in nasal polyp histopathology, may provide a more direct representation of the local inflammatory burden and could offer greater predictive value. Despite its potential, the role of eosinophilic cells/HPF as a predictor of dupilumab response remains underexplored in the literature.

To address this topic, we conducted a single-center, real-life cohort study to explore whether baseline eosinophilic cell density (cells/HPF) in nasal polyp tissue is associated with better clinical response to dupilumab in patients with CRSwNP, and to evaluate its potential as a histological marker guiding personalized treatment strategies.

## Materials and methods

This was a single-center retrospective observational study conducted at IRCCS Humanitas Research Hospital (Rozzano, Milan, Italy) between December 2020 and December 2024. The study protocol adhered to the ethical standards of the Declaration of Helsinki and was approved by the local Ethics Committee. Informed consent was obtained from all participants. Consecutive patients with refractory CRSwNP who were eligible for dupilumab therapy and consented to participate were enrolled. According to standard clinical practice in our department, the indication for monoclonal antibody (mAb) was established following a multidisciplinary board discussion involving rhinologists, allergologists, and pulmonologists. Eligibility criteria were in line with the requirements of the Italian Medicines Agency (Agenzia Italiana del Farmaco, AIFA, Roma, Italy) and included:

i. Severe disease, defined as a Nasal Polyp Score (NPS) ≥5 or a Sinonasal Outcome Test-22 (SNOT-22) score ≥50
ii. Failure or refusal of prior corticosteroid and/or surgical treatment.

**Fig. 1:**
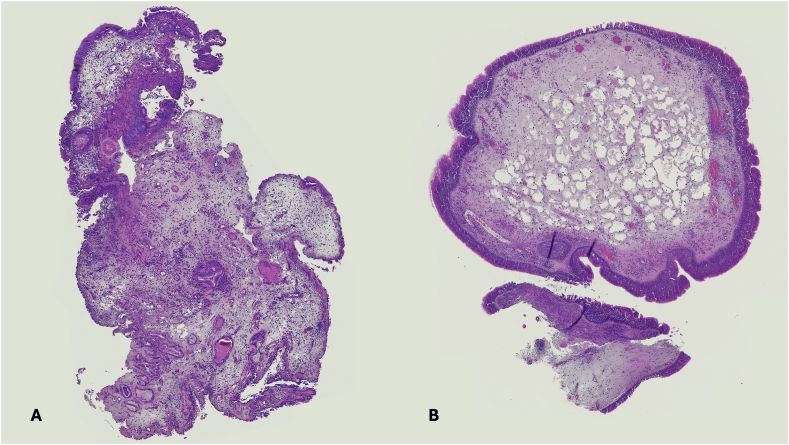
*legend:* Hematoxylin and eosin-stained section of nasal polyp tissue samples comparing different distributions and of inflammatory cells. A) High widespread and inhomogeneous inflammatory infiltration (>50 eos/HPF); B) Low and homogeneous inflammatory infiltration (<50 eos/HPF).

All enrolled patients had previously undergone at least 1 functional endoscopic sinus surgery (FESS), including bilateral maxillary antrostomy, anterior and posterior ethmoidectomy, sphenoidotomy, and frontal sinusotomy (eg, Draf IIa/b), with preservation of the physiological mucociliary drainage. Patients who underwent extended non-mucosa sparing approaches (eg, reboot surgery) were excluded. Additional exclusion criteria included age <18 years and unwillingness to participate. A baseline evaluation (T0) was performed 1 month prior to initiating dupilumab therapy. At this timepoint, a mucosal biopsy was obtained to assess tissue eosinophilic infiltration. Biopsies were taken from the posteromedial wall of the maxillary sinus, adjacent to the lateral ethmoid wall. Samples were fixed in formalin, embedded in paraffin, and sectioned at 3 μm thickness using a microtome. Hematoxylin and eosin staining was performed for histological assessment and eosinophil count (Fig. 1). Patients were instructed to discontinue intranasal medications for at least three weeks prior to biopsy. At baseline, patients underwent nasal endoscopy with assessment of Nasal Polyp Score (NPS), Lund-Kennedy Score (LKS) and were also required to fill in the Sinonasal Outcome Test-22 (SNOT-22). Furthermore, a CT-scan with assessment of Lund-Mackay Score (LMS) was performed for the assessment of sinonasal opacification. The dosage of dupilumab was 300 mg subcutaneously every 15 days in patients without severe asthma.^14^ Conversely, those who were in comorbidity for this condition were administered a 600 mg loading dose with subsequent administration of 300 mg injections biweekly, according to international guidelines.^15^ Topical intranasal medications (eg, nasal rinses and topical corticosteroids), were regularly pursued across the entire cohort. Objective outcomes (NPS, LKS, LMS) and patient-reported outcome measures (SNOT-22) were reassessed at 1 month (T1, except for LMS), 3 months (T2), and 12 months (T3) after treatment initiation. The minimum sample size required to assess improvements in single outcomes across the study period was calculated based on an α-error set of 0.05 and a β-error at 0.20, for a study power of 80%. Minimal clinically important differences (MCID) were defined based on previous literature as: NPS >1, LMS >5, and SNOT-22 > 8.9.^4^^,^^16^

Histological evaluation was performed using light microscopy. In accordance with published protocols,^17^ eosinophil infiltration was assessed by the pathologist at 20 × magnification, with attention to the distribution pattern (heterogeneous vs homogenous) and quantification of eosinophils per high-power field (HPF). The patients were then categorized into 2 groups based on eosinophilic infiltration: i) low infiltration: <50 eosinophils/HPF; ii) high infiltration: ≥50 eosinophils/HPF. In cases of heterogeneous eosinophil distribution, the classification was based on the highest count in the most representative field. Outcomes were compared between groups at each timepoint. Statistical analysis was conducted with SPSS Statistics for Macintosh, Version 28.0 (IBM Corp, Armonk, NY). Continuous variables were presented as mean, range and standard deviation, while discrete variables were expressed as absolute counts and percentages. Normal distribution was assessed trough *S*-*Wilk* test. Changes of each variable over timepoints was measured using two-way repeated measures analysis of variance (ANOVA). *Mauchly's* test was used to assess the assumption of sphericity (ie, homogeneity of the variances among groups); if violated, the *Greenhouse–Geisser* correction was applied. Within-subject effects assessed parameter changes over time, whereas between-subject effects evaluated whether baseline tissutal eosinophilic infiltration level influenced the outcomes in course of treatment. This model also included an interaction term to further investigate if differences in single outcomes were dependent on baseline eosinophilic infiltration. Pairwise analysis was conducted to compare scores at each timepoint. *Bonferroni* correction was applied in case of multiple comparisons. Correlation between dichotomous and continuous variables was assessed through binary logistic regression analysis.

## Results

### Overview

Eighty-six patients were enrolled in the study (males: 56/86, 65.1%; mean age: 56.51 ± 9.97 years, range: 27–84). Demographics and baseline characteristics are presented in Table 1. Most of included subjects were asthmatic (n = 64/86, 74.4%) or atopic (n = 48/86, 55.8%). Specifically, 41 (47.7%) and 36 (41.9%) patients were sensitized to seasonal and perennials allergens, respectively. Twenty-nine (n = 29/86, 33.7%) referred nonsteroidal anti-inflammatory drugs (NSAIDs) hypersensitivity. Overall, 24 patients (n = 24/86, 27.9%) were classified as affected by NSAID-Exacerbated Respiratory Disease (N-ERD). The average number of previous surgeries was 2.60 ± 3.12 (range: 1–19). No patients required oral corticosteroids nor additional sinus surgery during dupilumab treatment in order to control nasal symptoms.

**Table 1:** Patient's baseline characteristics. HPF: High power field; OCS: Oral corticosteroid; N-ERD: NSAID-exacerbated respiratory disease

		Overall (n = 86)	<50 eosinophils/HPF (n = 57)	≥50 eosinophils/HPF (n = 29)	p-value
Gender	Females (%)	30 (34.9)	23 (40.4)	7 (24.1)	0.104
	Males (%)	56 (65.1)	34 (59.6)	22 (75.9)	
Age	Mean (median)	56.51 (57.50)	57.70 (58.0)	54.17 (54.0)	0.113
Smoking	Yes (%)	24 (27.9)	16 (28.1)	8 (27.6)	0.962
	No (%)	62 (72.1)	41 (71.9)	21 (72.4)	
Comorbidities	Asthma (%)	64 (74.4)	44 (77.2)	20 (70.0)	0.283
	Severe asthma (%)	7 (8.13)	5 (8.7)	2 (6.9)	0.561
	Allergy (%)	48 (55.8)	35 (61.4)	13 (44.8)	0.109
	N-ERD (%)	24 (27.9)	14 (24.6)	10 (34.4)	0.332
Previous (1-year) OCS assumption (≥2 cycles)	Yes (%)	46 (53.5)	24 (42.1)	15 (51.7)	0.397
	No (%)	40 (46.5)	33 (57.9)	14 (48.3)	
Previous functional sinus surgeries	Mean (range)	2.60 (1–16)	2.44 (1–12)	2.93 (1–16)	0.558
Blood eosinophils	Mean (cells/μL) ± SD	302.42 ± 134.12	291.23 ± 182.86	324.43 ± 289.13	0.517
IgE-total	Mean (IU/mL) ± SD	393.63 ± 321.43	389.32 ± 321.74	402.11 ± 398.90	0.672

Tissue eosinophil infiltration was <50 cells/high-power field (HPF) in 57 patients (66.3%, M/F: 34/23, mean age: 57.70 ± 10.16) and ≥50 cells/HPF in the remaining 29 patients (33.7%; M/F: 22/7, mean age: 54.17 ± 9.32). There were no significant differences in demographics and baseline characteristics between the 2 groups (Table 1). At T0, mean blood eosinophil count was 302.42 ± 134.12 cells/μL (range: 100–600), and mean total serum IgE was 393.63 ± 321.43 IU/mL (range: 21.3–1789.0). No statistically significant differences in baseline hematochemical parameters were observed (mean difference: blood eosinophils = 33.20 ± 41.08 cells/μL, p = 0.517; total IgE = 12.79 ± 39.69 IU/mL, p = 0.672). Similarly, no correlation between circulating and tissue eosinophils was observed at regression analysis (p = 0.273). The distribution of objective and patient-reported outcomes followed a normal distribution according to the *Shapiro-Wilk* test (p > 0.05). The minimum sample size required to assess improvements in each parameter overtime was ≥22 and was hence achieved in our cohort. Table 2 displays single outcomes at various timepoints.

**Table 2:** Outcomes at each timepoint in the overall population and by eosinophilic infiltration subgroups. NPS: Nasal Polyp Score; LKS: Lund Kennedy Score; SNOT-22: Sinonasal Outcome Test-22; LMS: Lund Mackay Score; HPF: High Power Field

		Baseline (T0)	1 month (T1)	3 month (T2)	12 month (T3)
NPS	Overall	6.11 ± 1.69	4.24 ± 2.31	2.98 ± 2.20	2.04 ± 2.12
	<50 eosinophils/HPF	6.17 ± 1.63	3.89 ± 2.42	2.83 ± 2.06	2.03 ± 2.12
	≥50 eosinophils/HPF	6.00 ± 1.68	4.89 ± 2.00	3.26 ± 2.47	2.05 ± 2.17
LKS	Overall	10.15 ± 2.29	6.36 ± 3.78	6.59 ± 2.87	5.47 ± 3.03
	<50 eosinophils/HPF	10.00 ± 2.33	6.06 ± 3.77	6.39 ± 2.95	5.27 ± 2.99
	≥50 eosinophils/HPF	10.46 ± 2.23	7.00 ± 3.82	7.00 ± 2.70	5.88 ± 3.13
SNOT-22	Overall	63.20 ± 22.99	37.45 ± 21.06	25.61 ± 17.76	19.50 ± 14.67
	<50 eosinophils/HPF	61.59 ± 22.43	40.25 ± 22.53	30.66 ± 18.01	23.25 ± 14.97
	≥50 eosinophils/HPF	66.41 ± 17.81	31.86 ± 16.90	15.50 ± 12.29	12.00 ± 10.95
LMS	Overall	18.75 ± 4.32	–	11.18 ± 4.27	7.70 ± 4.16
	<50 eosinophils/HPF	18.76 ± 4.11		11.30 ± 4.43	7.38 ± 4.38
	≥50 eosinophils/HPF	18.71 ± 5.09		10.78 ± 3.91	8.64 ± 3.39

### Endoscopic parameters

At baseline, the mean endoscopic scores were NPS = 6.11 ± 1.69 (range: 2–8) and LKS = 10.15 ± 2.29 (range: 4–14; Table 2). By T1, we observed a significant improvement for both parameters in the overall population (mean difference: NPS = 1.87 ± 2.36, p < 0.001; LKS = 3.79 ± 3.90, p < 0.001; Table 3). At T2, a further significant reduction in NPS was observed compared to T1 (mean difference: 1.26 ± 1.61, p < 0.001). Finally, by T3, both NPS and LKS showed additional significant improvements relative to T2 (mean difference: NPS = 0.94 ± 1.32, p < 0.001; LKS = 1.12 ± 3.06, p = 0.026). The minimal clinically important difference for NPS was achieved at T1 and T2 in the overall population and by subgroups. At T3, only those with high eosinophilic infiltration at baseline still achieved the MCID, showing a more sustained effect in this sample (Table 3). Within-subjects ANOVA confirmed a significant improvement of endoscopic parameters overtime (NPS, LKS = p < 0.001). There was no significant interaction between the degree of eosinophilic infiltration and changes in endoscopic scores in course of treatment (NPS: p = 0.145; LKS: p = 0.465). Subgroup analysis revealed no significant differences in mean postoperative outcomes between patients with high versus low baseline tissue eosinophilia (Table 3). Similarly, between-subjects effects analysis confirmed that baseline eosinophilic infiltration did not significantly influence endoscopic outcomes (NPS: p = 0.518; LKS: p = 0.201).

**Table 3:** Pairwise comparison of mean outcomes at each timepoint. Bonferroni correction was applied. ϕ = p < 0.05. η = baseline (T0) versus 3-months (T2). NPS: Nasal Polyp Score; LKS: Lund Kennedy Score; SNOT-22: Sinonasal Outcome Test-22; LMS: Lund Mackay Score; HPF: High Power Field.

		T0 vs T1	T1 vs T2	T2 vs T3
NPS	Overall	1.87 ± 2.36^ϕ^	1.26 ± 1.61^ϕ^	0.94 ± 1.32^ϕ^
	<50 eosinophils/HPF	2.28 ± 2.50^ϕ^	1.06 ± 1.47^ϕ^	0.80 ± 1.42^ϕ^
	≥50 eosinophils/HPF	1.11 ± 1.95^ϕ^	1.63 ± 1.85^ϕ^	1.21 ± 1.05^ϕ^
LKS	Overall	3.79 ± 3.90^ϕ^	−0.23 ± 0.98	1.12 ± 3.06^ϕ^
	<50 eosinophils/HPF	3.94 ± 3.71^ϕ^	−0.33 ± 0.78	1.12 ± 3.07^ϕ^
	≥50 eosinophils/HPF	3.46 ± 4.30^ϕ^	0.00 ± 0.85	1.12 ± 3.11^ϕ^
SNOT-22	Overall	25.75 ± 23.07^ϕ^	11.84 ± 13.06^ϕ^	6.11 ± 12.56^ϕ^
	<50 eosinophils/HPF	21.34 ± 24.90^ϕ^	9.59 ± 14.30^ϕ^	7.41 ± 14.35^ϕ^
	≥50 eosinophils/HPF	34.55 ± 15.92^ϕ^	16.36 ± 11.89^ϕ^	3.50 ± 7.46^ϕ^
LMS	Overall	7.57 ± 4.80^ηϕ^	–	3.48 ± 4.31^ϕ^
	<50 eosinophils/HPF	7.46 ± 5.19^ηϕ^		3.92 ± 4.41^ϕ^
	≥50 eosinophils/HPF	7.93 ± 3.91^ηϕ^		2.14 ± 3.82^ϕ^

### Sinonasal outcome Test-22

**Fig. 2:**
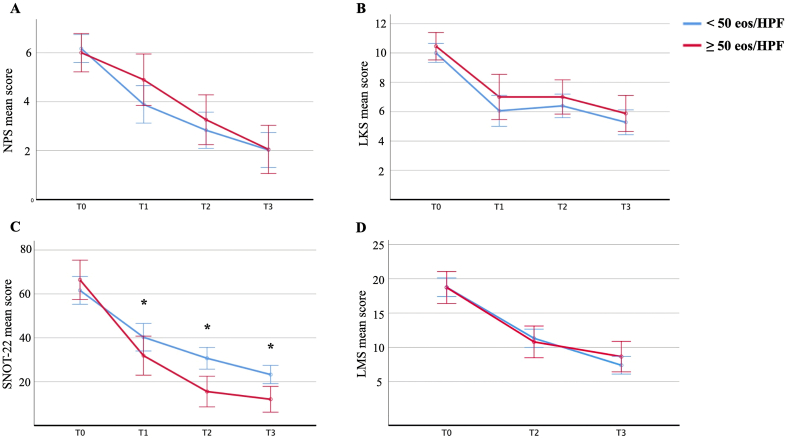
Trend of single outcomes at each timepoint by baseline eosinophilic infiltration. Blue line: <50 eosinophils/HPF; Red line: ≥50 eosinophils/HPF. ∗ = p < 0.05; NPS: Nasal Polyp Score; LKS: Lund Kennedy Score; SNOT-22: Sinonasal Outcome Test-22; LMS: Lund Mackay Score

Prior to dupilumab initiation, patients exhibited a considerable symptom burden, with a mean SNOT-22 score of 63.20 ± 22.99 (range: 23–95; Table 2). No differences were observed according to eosinophilic infiltration (mean difference = 4.82 ± 4.56, p = 0.519). Similarly, no significant correlation was found between the overall SNOT-22 score and eosinophilic infiltration (p = 0.515). By T1, there was a significant improvement of the mean SNOT-22 (mean difference = 25.75 ± 23.07, p < 0.001), which was sustained through subsequent evaluations (mean difference: T1 vs T2 = 11.84 ± 13.06, p < 0.001; T2 vs T3 = 6.11 ± 12.56, p < 0.001; Table 3). The minimal clinically important difference was overwhelmed at both T1 and T2; however, the change from T2 to T3 did not meet the threshold, indicating stabilization of symptom improvement in both subgroups (Table 3). Within-subjects effects analysis confirmed a significant reduction of the SNOT-22 over timepoints (p < 0.001). A significant interaction was observed between baseline tissue eosinophilic infiltration and changes in SNOT-22 (p = 0.001). Specifically, patients with high eosinophilic infiltration demonstrated significantly lower SNOT-22 scores at each follow-up examination compared to those with lower eosinophil counts (mean difference: T1 = 3.40 ± 5.03, p = 0.042; T2 = 6.34 ± 3.41, p < 0.001; T3 = 3.44 ± 3.09, p = 0.001; Fig. 2). Between-subjects ANOVA further confirmed that baseline eosinophilic infiltration significantly influenced the improvement trajectory of SNOT-22 scores overtime (p = 0.045).

### Sinus opacification

At baseline, the mean LMS was 18.75 ± 4.32 (range: 9–24; Table 2). By T2, a significant reduction in the overall sinus opacification was observed compared to T0 (mean difference: 7.57 ± 4.80, p < 0.001). This improvement continued at T3, with a further significant decrease in LMS (mean difference: 3.48 ± 4.31, p < 0.001; Table 3), proving a sustained effectiveness of treatment on sinonasal inflammation overtime. The minimal clinically important difference was achieved at T2 compared to baseline, although the improvements from T2 to T3 did not meet the MCID threshold in both the overall population and by subgroups. Within-subjects effects ANOVA confirmed a significant reduction in mean LMS across timepoints (p < 0.001). Nevertheless, the interaction between baseline eosinophil infiltration and changes in sinonasal opacification score was not significant (p = 0.293). Pairwise comparison showed no significant differences in LMS across groups at each timepoint (mean difference: T2 = 0.45, p = 0.161; T3 = 0.07, p = 0.868). Between-subjects analysis confirmed lack of significant effect of baseline tissue eosinophils on radiologic improvement over time (p = 0.819).

## Discussion

Biologic therapies have significantly expanded treatment options for patients with CRSwNP, especially those with persistent symptoms despite conventional care.^18^ By targeting key inflammatory pathways, these agents enable a more precise, endotype-oriented approach that goes beyond traditional symptom control.^7^ By selectively modulating key pathways of type 2 inflammation, biologics present as a valuable option for CRSwNP patients who are refractory to conventional treatment. These agents often reduce the need for revision sinus surgery, frequently complicated by altered sinonasal anatomy or extensive polyposis,^19^ and help minimizing systemic corticosteroid use, associated with well documented long-term adverse effects.^20^ Also, they have demonstrated excellent efficacy on symptom burden and quality of life, all while maintaining a very favorable safety profile.^21^

Despite their demonstrated benefits, some considerations remain regarding the use of biologic therapies. Their high cost raises ongoing discussions about cost-effectiveness.^22^ In parallel, long-term safety data are still being gathered and will be important to confirm their favorable risk profile over extended periods of time.^18^^,^^23^ Finally, clinical response is not uniform, and a subset of patients may still require systemic corticosteroids or revision surgery. According to current literature, approximately 10% of patients treated with dupilumab eventually require rescue therapy, underscoring the need for better identification of predictive markers of response.^3^ Several studies have attempted to identify reliable predictors of response to biologic therapy in CRSwNP; however, no single biomarker has been definitively established, and the available evidence remains limited and heterogeneous.^9^^,^^10^^,^^16^

In our study, dupilumab confirmed its rapid and effective therapeutic action in patients with CRSwNP. In addition, our retrospective, single-center analysis provided new evidence supporting the possible predictive role of local tissue eosinophilia in enhancing clinical response to dupilumab. Specifically, we identified a statistically significant improvement in patient-reported symptoms, as measured by the SNOT-22 questionnaire, in patients with baseline eosinophilic infiltration ≥50 cells/HPF, compared to those with lower counts. This effect was already detectable at one month and was maintained throughout the 12-month follow-up period (Fig. 2).

De Corso et al previously reported a significant association between tissue eosinophil levels and disease severity in patients with CRSwNP.^24^ However, in our sample this association was not evident, as no significant differences in SNOT22 were reported. To our knowledge, no prior study has showed a significant relationship between baseline tissue eosinophilia and meaningful clinical improvement in symptom burden following treatment with dupilumab. This observed association may reflect a more pronounced type 2 inflammatory environment, characterized by elevated tissue eosinophils and sustained activation of IL-4 and IL-13 pathways, which are directly targeted by dupilumab.

Our results are, also, consistent with those of Kim et al, which showed that higher eosinophil counts in nasal polyp tissue were associated with improved clinical outcomes following treatment in CRSwNP patients with comorbid asthma.^25^ Similarly, Lou et al reported that high tissue eosinophil levels predicted polyp recurrence in Chinese patients, suggesting that local eosinophilic infiltration may indicate a more active and clinically meaningful disease subtype.^26^

In our cohort, no significant differences were observed between groups in objective endoscopic (NPS, LKS) or radiologic (LMS) scores. Interestingly, although the overall change in NPS did not reach statistical significance between groups, only patients with high baseline tissue eosinophilia achieved the MCID at 12 months, suggesting a more sustained effect in this subgroup. While a clear association emerged between tissue eosinophilia and patient-reported outcomes, this correlation was not reflected in structural measures.^18^ This may indicate a partial dissociation between symptom perception and morphological changes. In the context of a systemic inflammatory disease, relying solely on polyp size or radiologic findings to evaluate treatment response in patients with CRSwNP may fail to capture the full clinical trajectory of the underlying inflammatory process. Although standardized clinical evaluation is essential, its accuracy may be limited if patient heterogeneity is overlooked. In this light, the limitations of current objective outcome measures become evident, as they rely primarily on gross anatomical rather than on microscopic changes induced by IL-4 and IL-13 blockage, such as reduced mucus production, improved cell contractility, and restored B-cell class switching.^21^ Implementing microscopic analysis in clinical practice could help overcome these issues.

For this study, we included a homogeneous cohort of patients treated with dupilumab, based on strict inclusion criteria. Baseline characteristics did not differ significantly between groups (Table 1). The minimum required sample size was met to assess changes in each parameter across timepoints. Importantly, baseline SNOT-22 scores were comparable between groups; however, longitudinal trends varied according to baseline tissue eosinophilia (Fig. 1), supporting the reliability of our findings.

The choice of 50 cells/HPF as a cut-off is supported by previous studies that have proposed this threshold as a clinically meaningful tool for stratifying inflammatory sinonasal disease based on tissue eosinophil density.^17^^,^^27^, ^28^, ^29^ We acknowledge that using this threshold resulted in a slight numerical imbalance between the two groups. Nevertheless, baseline characteristics were homogeneous, thereby supporting the validity of subsequent comparative analyses (Table 1). Also, the simplicity and ease of application of this approach enhance its potential utility in routine histopathological assessment. At the same time, the heterogeneous distribution of eosinophils within polyp tissue and possible interobserver variability suggest that additional or alternative metrics could improve consistency across studies. These may include the proportion of eosinophils relative to total inflammatory cells or absolute counts per unit area. It would also be of interest to investigate how eosinophils are spatially organized within the inflammatory microenvironment, which may provide further insights into disease activity and treatment response.^30^

While these insights are promising, several limitations of our study should be considered. Even though the minimum sample size was achieved, the retrospective nature of the study and the relatively small cohort may limit the generalizability of the results. Additionally, being conducted in a single-center setting, the study may be subject to selection bias. Although our findings support the potential of tissue eosinophilia as a predictive marker of dupilumab response, larger prospective studies are needed to confirm whether eosinophil-rich patients consistently benefit more. To strengthen the evidence, further analyses are required to evaluate the impact of tissue eosinophils on patient-reported outcomes beyond rhinologic symptoms, using validated tools specifically designed for this purpose. Future studies should also determine whether tissue eosinophilia predicts treatment outcomes in patients receiving different monoclonal antibodies targeting type 2 inflammation (e.g., anti–IL-5, anti-IgE). In addition, research should examine the reproducibility of the 50 cells/HPF threshold and define its role in personalized management of CRSwNP. Finally, standardization of eosinophil quantification methods and consensus in histopathologic reporting are needed to facilitate broader clinical application. While eosinophilic cells per HPF represents a practical and widely accessible metric, alternative approaches such as calculating absolute eosinophil counts per unit area or assessing their proportion relative to total inflammatory cells may provide a more detailed understanding of local inflammatory activity.

## Conclusion

Dupilumab confirmed its well-established efficacy in patients with CRSwNP, with a rapid onset of action and an excellent safety profile. Our results suggest that the extent of tissue eosinophilic infiltration may be associated with enhanced clinical improvement in CRSwNP patients treated with dupilumab. These findings strengthen the potential role of tissue-based inflammatory profiling in guiding therapeutic decisions. Incorporating histopathological evaluation into the clinical workflow could contribute to more informed patient selection and support the development of personalized treatment strategies.

## Consent to publish

Informed consent was obtained from all subjects involved in the study.

## Ethics statement

The study was adherent to the ethical standards of the Declaration of Helsinki and was approved by the local Ethics Committee (ref/ICH/3402).

## Authors contribution

Conceptualization, Giorgio Walter Canonica, Enrico Heffler, Giovanni Paoletti; Methodology, Barbara Fiamengo, Silvia Uccella; Validation, Francesca Puggioni; Formal analysis, Michele Cerasuolo; Investigation, Camilla Zimello, Luca Cerri, Giulia Mari; Supervision, Giuseppe Spriano. Giuseppe Mercante; Project administration, Luca Malvezzi; Writing—original draft preparation, Gian Marco Pace; Writing—review and editing, Francesco Giombi. All authors have read and agreed to the published version of the manuscript.

## Declaration of competing interest

The authors report no competing interests.

## References

[1] Fokkens W.J., Lund V.J., Hopkins C. (2020). European position paper on rhinosinusitis and nasal polyps 2020. Rhinology.

[2] De Corso E., Baroni S., Settimi S. (2022). Sinonasal biomarkers defining type 2-high and type 2-low inflammation in chronic rhinosinusitis with nasal polyps. J Personalized Med.

[3] Bachert C., Han J.K., Desrosiers M. (2019). Efficacy and safety of dupilumab in patients with severe chronic rhinosinusitis with nasal polyps (LIBERTY NP SINUS-24 and LIBERTY NP SINUS-52): results from two multicentre, randomised, double-blind, placebo-controlled, parallel-group phase 3 trials. Lancet.

[4] Fokkens W., Trigg A., Lee S.E. (2023). SYNAPSE study group. Mepolizumab improvements in health-related quality of life and disease symptoms in a patient population with very severe chronic rhinosinusitis with nasal polyps: psychometric and efficacy analyses from the SYNAPSE study. J Patient-Rep Outcomes.

[5] Gevaert P., Omachi T.A., Corren J. (2020). Efficacy and safety of omalizumab in nasal polyposis: 2 randomized phase 3 trials. J Allergy Clin Immunol.

[6] Bachert C., Han J.K., Desrosiers M.Y. (2022). Efficacy and safety of benralizumab in chronic rhinosinusitis with nasal polyps: a randomized, placebo-controlled trial. J Allergy Clin Immunol.

[7] Rodriguez-Iglesias M., Calvo-Henríquez C., Martin-Jimenez D. (2025). Effect of dupilumab in CRSwNP sinonasal outcomes from real life studies: a systematic review with meta-analysis. Curr Allergy Asthma Rep.

[8] Chuang C.C., Guillemin I., Bachert C. (2022). Dupilumab in CRSwNP: responder analysis using clinically meaningful efficacy outcome thresholds. Laryngoscope.

[9] Pace G.M., Giombi F., Pirola F. (2025). Prediction of clinical response to dupilumab for CRSwNP based on the Amsterdam classification of completeness of endoscopic sinus surgery (ACCESS) score. Ann Otol Rhinol Laryngol.

[10] Dorismond C., Krysinski M.R., Trivedi Y. (2025). Real-world predictors of dupilumab prescription in patients with chronic rhinosinusitis with nasal polyps. Int Forum Allergy Rhinol.

[11] Ogulur I., Mitamura Y., Yazici D. (2025). Type 2 immunity in allergic diseases. Cell Mol Immunol.

[12] Fujieda S., Matsune S., Takeno S. (2022). Dupilumab efficacy in chronic rhinosinusitis with nasal polyps from SINUS-52 is unaffected by eosinophilic status. Allergy.

[13] Feng T., Li T., Cao W. (2021). Peripheral blood eosinophil levels in chronic rhinosinusitis and its predictive value in eosinophilic chronic rhinosinusitis. Acta Otolaryngol.

[14] Chung K.F., Wenzel S.E., Brozek J.L. (2014). International ERS/ATS guidelines on definition, evaluation and treatment of severe asthma. Eur Respir J.

[15] Bateman E.D., Hurd S.S., Barnes P.J. (2008). Global strategy for asthma management and prevention: GINA executive summary. Eur Respir J.

[16] Han J.K., Bachert C., Lee S.E. (2022). Estimating clinically meaningful change of efficacy outcomes in inadequately controlled chronic rhinosinusitis with nasal polyposis. Laryngoscope.

[17] McHugh T., Snidvongs K., Xie M. (2018). High tissue eosinophilia as a marker to predict recurrence for eosinophilic chronic rhinosinusitis: a systematic review and meta-analysis. Int Forum Allergy Rhinol.

[18] Giombi F., Pace G.M., Nappi E. (2024). Radiological versus clinical 1-year outcomes of dupilumab in refractory CRSwNP: a real-life study. Laryngoscope.

[19] Pfaar O., Peters A.T., Taillé C. (2025). Chronic rhinosinusitis with nasal polyps: key considerations in the multidisciplinary team approach. Clin Transl Allergy.

[20] Canonica G.W., Colombo G.L., Bruno G.M., SANI Network (2019). Shadow cost of oral corticosteroids-related adverse events: a pharmacoeconomic evaluation applied to real-life data from the Severe Asthma Network in Italy (SANI) registry. World Allergy Organ J.

[21] Kratchmarov R., Dharia T., Buchheit K. (2025). Clinical efficacy and mechanisms of biologics for chronic rhinosinusitis with nasal polyps. J Allergy Clin Immunol.

[22] Brown W.C., Senior B. (2020). A critical look at the efficacy and costs of biologic therapy for chronic rhinosinusitis with nasal polyposis. Curr Allergy Asthma Rep.

[23] Chen H., Wang L., Zhang J. (2025). Long-term efficacy and safety of different biologics in treatment of chronic rhinosinusitis with nasal polyps: a network meta-analysis. Braz J Otorhinolaryngol.

[24] De Corso E., Corbò M., Montuori C. (2025). Blood and local nasal eosinophilia in chronic rhinosinusitis with nasal polyps: prevalence and correlation with severity of disease. Acta Otorhinolaryngol Ital.

[25] Kim M.K., Cho S.H., Lee H.N. (2024). Significance as a prognostic factor of eosinophil count in nasal polyp tissue in patients with chronic rhinosinusitis accompanied by asthma. J Clin Med.

[26] Lou H., Meng Y., Piao Y. (2015). Predictive significance of tissue eosinophilia for nasal polyp recurrence in the Chinese population. Am J Rhinol Allergy.

[27] Ma L., Shi J., Wang K. (2022). Clinical characteristics of patients with CRSwNP with intensely high eosinophil level. Laryngoscope Investig Otolaryngol.

[28] Song W., Wang C., Zhou J. (2017). IL-33 expression in chronic rhinosinusitis with nasal polyps and its relationship with clinical severity. ORL J Otorhinolaryngol Relat Spec.

[29] Baba S., Kondo K., Kanaya K. (2014). Expression of IL-33 and its receptor ST2 in chronic rhinosinusitis with nasal polyps. Laryngoscope.

[30] Erjefält J.S. (2025). Spatial eosinophil phenotypes as immunopathogenic determinants in inflammatory diseases. Cells.

